# A New Perspective on Transcriptional System Regulation (TSR): Towards TSR Profiling

**DOI:** 10.1371/journal.pone.0001656

**Published:** 2008-02-20

**Authors:** Rudolf S. N. Fehrmann, Hendrik J. M. de Jonge, Arja ter Elst, André de Vries, Anne G. P. Crijns, Alida C. Weidenaar, Frans Gerbens, Steven de Jong, Ate G. J. van der Zee, Elisabeth G. E. de Vries, Willem A. Kamps, Robert M. W. Hofstra, Gerard J. te Meerman, Eveline S. J. M. de Bont

**Affiliations:** 1 Department of Genetics, University Medical Center Groningen, University of Groningen, Groningen, The Netherlands; 2 Division of Pediatric Oncology, Department of Pediatrics, Beatrix Children's Hospital, University Medical Center Groningen, University of Groningen, Groningen, The Netherlands; 3 Department of Gynecology, University Medical Center Groningen, University of Groningen, Groningen, The Netherlands; 4 Department of Medical Oncology, University Medical Center Groningen, University of Groningen, Groningen, The Netherlands; Lehigh University, United States of America

## Abstract

It has been hypothesized that the net expression of a gene is determined by the combined effects of various transcriptional system regulators (TSRs). However, characterizing the complexity of regulation of the transcriptome is a major challenge. Principal component analysis on 17,550 heterogeneous human microarray experiments revealed that 50 orthogonal factors (hereafter called TSRs) are able to capture 64% of the variability in expression in a wide range of experimental conditions and tissues. We identified gene clusters controlled in the same direction and show that gene expression can be conceptualized as a process influenced by a fairly limited set of TSRs. Furthermore, TSRs can be linked to biological functions, as we demonstrate a strong relation between TSR-related gene clusters and biological functionality as well as cellular localization, *i.e.* gene products of similarly regulated genes by a specific TSR are located in identical parts of a cell. Using 3,934 diverse mouse microarray experiments we found striking similarities in transcriptional system regulation between human and mouse. Our results give biological insights into regulation of the cellular transcriptome and provide a tool to characterize expression profiles with highly reliable TSRs instead of thousands of individual genes, leading to a >500-fold reduction of complexity with just 50 TSRs. This might open new avenues for those performing gene expression profiling studies.

## Introduction

Biological systems have a layered complexity and it is known that a cell's activity is modulated by a network of co-regulated gene clusters.[1] Such modules are characterized by clusters of transcriptionally correlated genes, most often with related functions.[2] A number of studies using clustering algorithms based on similar expression patterns provided valuable clues about which strongly expressed genes are co-regulated in a small, specific set of experimental conditions.[1]–[3]

However, clustering algorithms are less effective when applied to large datasets of heterogeneous material. Basic clustering algorithms assign each gene to a single cluster of co-regulated genes, whereas it is hypothesized that the net expression of a gene is determined by the combined effects of various transcriptional system regulators (TSRs).[4]–[6] In addition, each level of transcriptional regulation may only be active in certain phenotypes and the remaining phenotypes will contribute to noise.[6] In contrast, principal component analysis (PCA) on a large heterogeneous set could enable us to use correlation structures of not only strong but also weakly expressed genes and could provide a global picture of the dynamics of gene expression on various transcriptional regulation levels. It could allow individual genes to be classified into groups that are similarly controlled by a specific TSR.

Unraveling the complexity of regulation of the transcriptome is a major challenge; as in principle an infinite number of TSRs could be needed to control the expression of thousands of genes ultimately leading to the large diversity seen in cellular phenotypes. In this study we identified a structure of transcriptional regulation by analyzing 17,550 heterogeneous microarray experiments. We found that the number of orthogonal factors needed to explain most of the variability in expression is fairly limited, even in a wide range of experimental conditions, tissues and even across species. Furthermore, using several different models, we show that these TSRs have biological relevance and yield reliable summary measurements of gene expression that are applicable to different tissue types as well as organisms.

## Results

### Transcriptional system regulators

**Figure 1:**
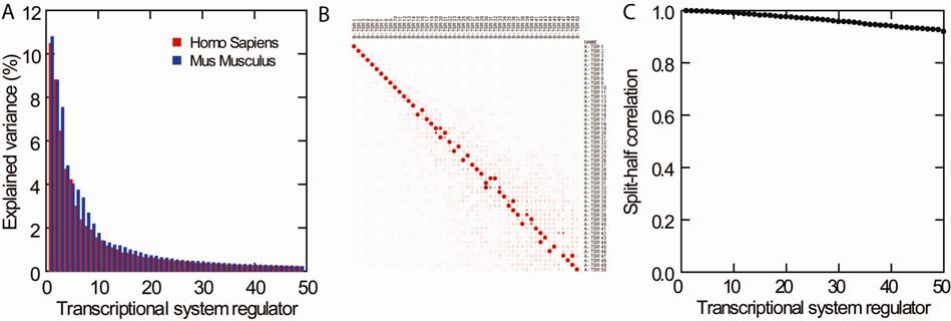
Explained variance and reliability of the first 50 transcriptional system regulators (TSRs). Panel A shows the explained variance for the first 50 TSRs in human and mouse. The percentage explained variance is depicted for each TSR. The cumulative percentage explained variance for the first 50 TSRs is 64% in human and 71% in mouse. Panel B shows a heat map where each box represents the Pearson correlation coefficient between TSRs generated from sets A and B, each using half of the data. Correlation coefficients of 0 and 1 are represented by black and red, respectively. Panel C shows the split-half correlations for the first 50 TSRs. (A TSR is a weighted sum of genes and a so-called TSR score can be calculated for each sample. In the split-half method, genes of a TSR score are split into two random parts and the resulting partial TSR scores are correlated (Pearson).)

Insight into the complexity of the regulation of the transcriptome was revealed by PCA on the expression correlation matrix of 13,032 genes in 17,550 human miscellaneous expression arrays. PCA demonstrated that 64% of the variance in expression of 13,032 genes was explained by only 50 orthogonal factors, called TSRs, which means a >500-fold reduction in complexity (Fig. 1A). Similar results were observed in mice where 50 TSRs explained 71% of the variance in expression of 9,062 genes in 3,934 arrays (Fig. 1A). Moreover, Figure 1A shows that the pattern of the percentage explained variance per TSR is highly similar between human and mouse. Tables S1 and S2 give factor loadings for the first 50 TSRs in human and mouse, respectively.

### Reliability of TSRs

To evaluate whether the identified TSRs depend on the specific set of selected microarray experiments, the human microarray data were randomly split into two halves and then two sets (A and B) of TSRs were generated, each using only half of the samples. Figure 1B contains a heat map showing correlation coefficients between TSRs generated in sets A and B. TSR1 generated in set A and TSR1 in set B correlated significantly (*R* = .999; *P*<1.0×10^−16^), indicating highly similar control of identical genes by TSR1 in both sets. Furthermore, the diagonal line in Figure 1B shows that TSRs generated in both sets were highly similar in their control of identical genes. For a few TSRs, the relative position in the order they were found was seen to be switched, but the same directions of variation were identified. These results indicate that the TSRs were reliably identified and were not artifacts due to sample selection. To further investigate whether the identified TSRs were not artifacts due to gene selection, we applied the split-half method to each TSR. Figure 1C shows the split-half correlations for the first 50 TSRs, which were high (>0.91), indicating their high internal consistency. In sum, the identified TSRs were robust and not artifacts due to selection of genes and/or tissues.

### Biological significance of TSRs

**Figure 2:**
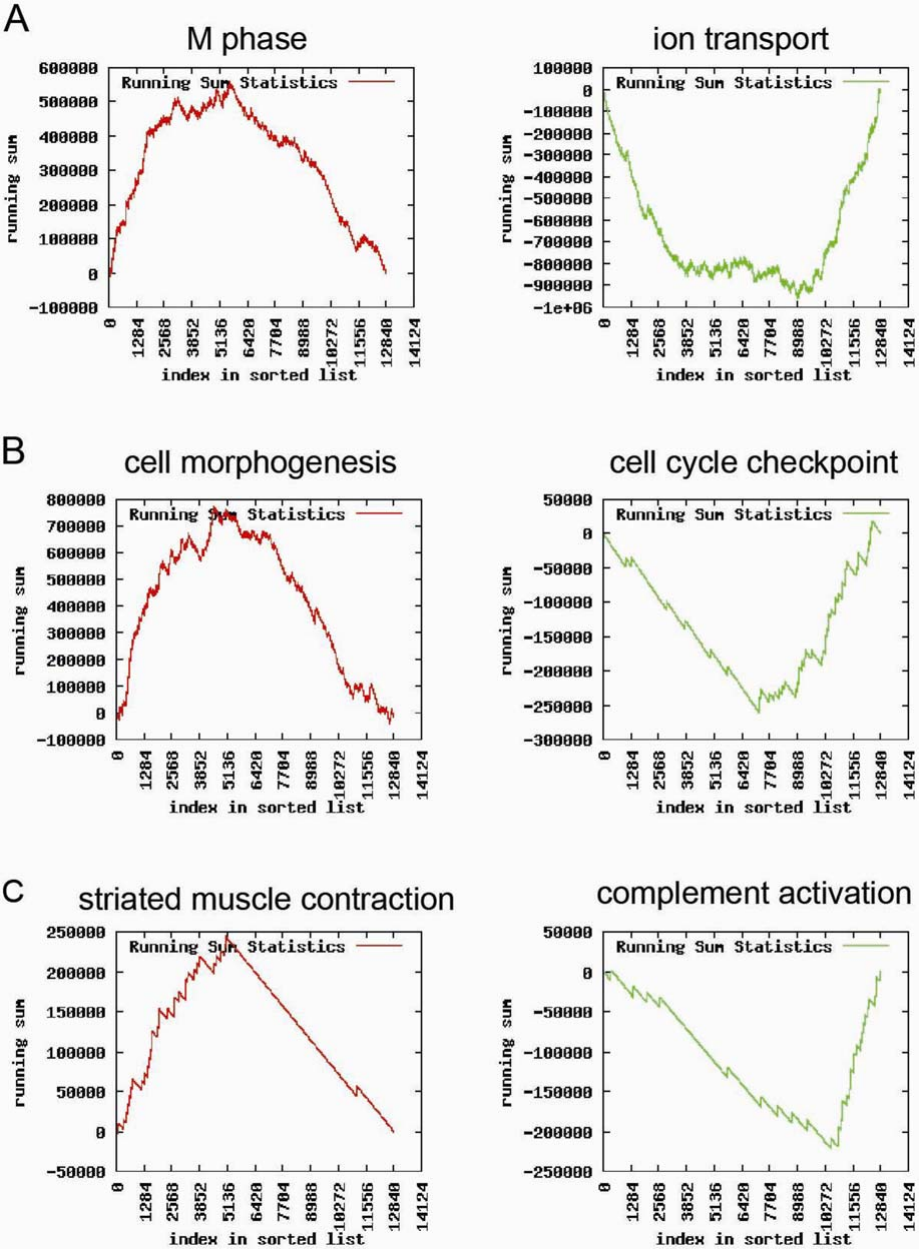
Biological significance of the transcriptional system regulators (TSRs). The most significant biological processes for either side of the respective TSR according to the GO ontology classification are shown for TSR1, TSR2 and TSR50 in panels A, B and C, respectively. Graphs depict the running sum statistics when applying gene set enrichment analysis. The running sum (*y*-axis) is shown as a function of the index in the list (*x*-axis) of genes ranked in ascending order according to their factor loadings within TSR. The red vs. green graphs show the biological coherence of opposing regulated gene clusters controlled by TSRs.

**Figure 3:**
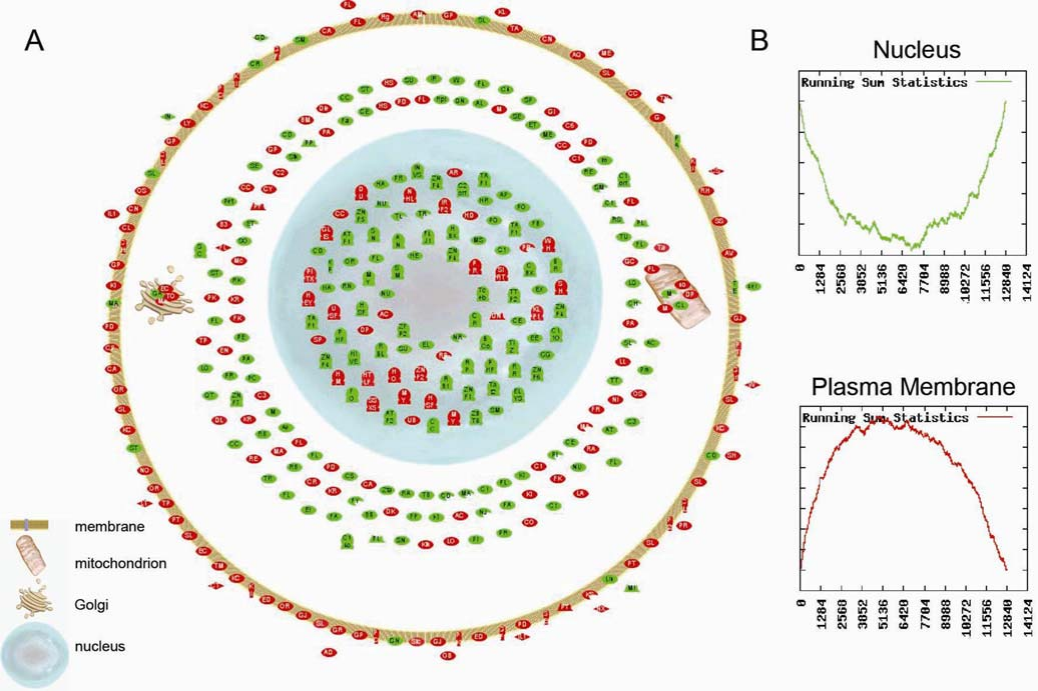
Relationship between cellular localization of genes controlled in opposite directions by TSR1. Panel A shows the cellular localization of the 400 most strongly controlled genes (highest factor loadings). The colors represent genes with positive (green) and negative (red) factor loadings. Panel B shows the results of gene set enrichment analysis with regard to cellular localization of the gene products. Graphs show the running sum statistics when applying gene set enrichment analysis. The running sum (*y*-axis) is shown as a function of the index in the list (*x*-axis) of genes ranked in ascending order according to their factor loadings within a TSR.

To validate that the identified TSRs are not merely mathematical constructs but contain biological coherence and to gain more biological insight into the regulation of the transcriptome, we performed Gene Set Enrichment Analysis (GSEA). The hypothesis is that our identified TSRs are related to known biologically related gene clusters represented by GO ontologies. Here we describe the GSEA results for the first, second and fiftieth TSR as examples. GSEA results for the first 25 TSRs are available in the supplementary data online. Among the genes most strongly influenced by TSR1, TSR2 and TSR50, many GO ontologies were significantly enriched (n = 488, n = 1157 and n = 119, respectively). Figure 2 shows the most significant biological processes per TSR according to the GO ontology classification on either side of it. The graphs show the enrichment score as a function of the index in the list (*x*-axis) of genes ranked according to the correlation between their expression and a TSR score (factor loading). Red graphs with a ‘mountain-like shape’ illustrate a specific GO ontology predominantly containing top ranked genes. In contrast, green graphs with a ‘valley-like shape’ illustrate a specific GO ontology predominantly containing bottom ranked genes. When genes belonging to a specific GO ontology are not top or bottom ranked but randomly distributed in the ranked list the graph will have a ‘zigzag’ shape. Furthermore, Figure 2 shows that TSRs have the capacity to influence the expression of genes involved in specific biological processes in opposite directions, *e.g.* TSR1 regulates genes belonging to GO ontology ‘progression through M phase’ *vs.* ‘ion transport’, TSR2 genes belonging to GO ontology ‘cell cycle checkpoint’ *vs.* ‘the cell morphogenesis’ and TSR50 genes belonging to GO ontology ‘striated muscle contraction’ *vs.* ‘complement activation’. Biological processes represented by GO ontologies can theoretically be influenced by more then one TSR; the contrast seen in TSR1 ‘progression through M phase’ *vs.* ‘ion transport’ was also seen in TSR3 for example. All enrichments for GO ontologies representing these six biological processes were highly significant; *P*<10^−8^. In addition, the encoded proteins of genes controlled in opposite directions (*i.e*. top vs. bottom ranked genes) by a TSR are generally located in other compartments of a cell (*e.g.* Golgi, mitochondrion, nucleus, etc.), as shown for TSR1 in Figure 3 for example. Panel A shows that the encoded proteins of the top-ranked genes of TSR1 (the limit of 200 was arbitrarily chosen) are generally located within the plasma membrane, whereas the 200 bottom-ranked genes of TSR1 are generally located within the nucleus of the cell. This is also visualized in Panel B, where the green ‘valley-like shape’ graph shows that the GO ontology for cellular localization ‘nucleus’ is enriched among the bottom-ranked genes. In contrast the red ‘mountain-like shape’ graph shows the enrichment of the GO ontology ‘plasma membrane’ at the top-ranked genes.

### Similarities in transcriptional system regulation between human and mouse

PCA on the combined two-species dataset consisting of 3,934 human and 3,934 mouse arrays was performed to assess the similarity of the structure in transcription regulation between human and mouse. PCA revealed that again 50 principal components (PCs) explained ∼73% of the total variance in combined human and mouse gene expression. In this specific two-species dataset the first PC (PC1) explained ∼25% of the total variance in expression. The distribution of PC1 scores for human samples showed no overlap with PC1 scores for mouse samples, suggesting that PC1 summarizes the variation in expression caused by species differences (Figure S1). Biological processes such as RNA processing, ion transport and primary metabolism were enriched in PC1 using GSEA analysis. Except for PC1, all PCs showed a strong overlap between human and mouse PC scores, suggesting that gene expression in human and mouse is similarly influenced by regulatory processes influencing evolutionary related gene clusters in the same direction.

### Mapping human and mouse TSRs

**Figure 4:**
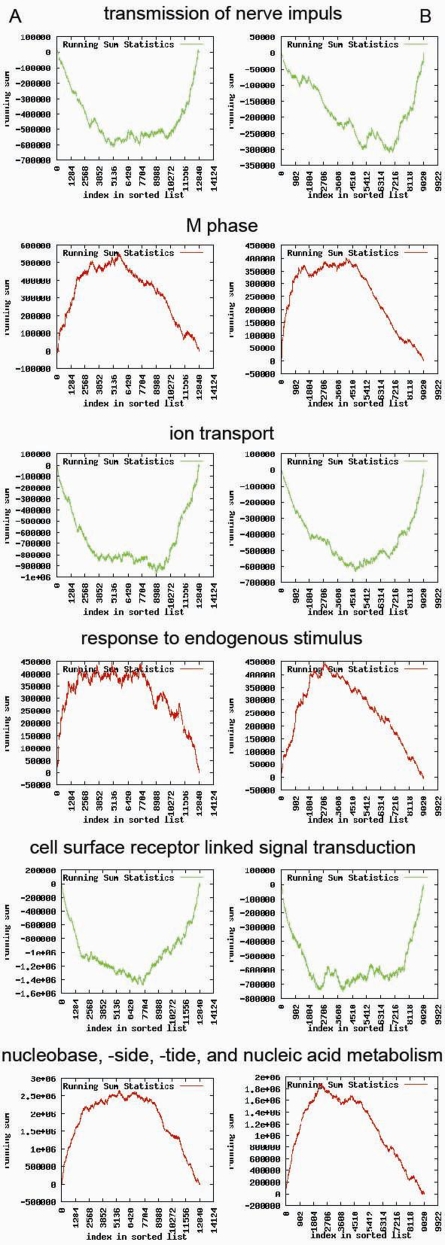
Similarities in transcriptional system regulation between human and mouse. Panels A and B show the results of gene set enrichment analysis for six biological processes for human TSR1 and mouse TSR2, respectively. Graphs show the running sum statistics. The running sum (*y*-axis) is shown as a function of the index in the list (*x*-axis) of genes ranked in ascending order according to their factor loadings with a TSR. The red vs. green graphs show the biological coherence of regulated gene clusters controlled by TSRs in both species.

The results above suggest a high similarity in the structure of transcriptional regulation between human and mouse. To assess similarities in regulation of biological processes, we mapped TSRs generated in the human dataset to TSRs generated in the mouse dataset. The Spearman rank correlation coefficients between human and mouse TSRs showed that human TSR1 was most strongly correlated with mouse TSR2 (*R* = .489, *P* = 1.0×10^−6^). Correlation coefficients between the first 25 human and mouse TSRs are given in Table S3. As an example of the strong resemblance in transcriptional system regulation between human and mouse, Figure 4 shows that identical biological processes are enriched and similarly controlled in one direction (human TSR1 *vs*. mouse TSR2).

### Regional control of chromosomal domains

**Figure 5:**
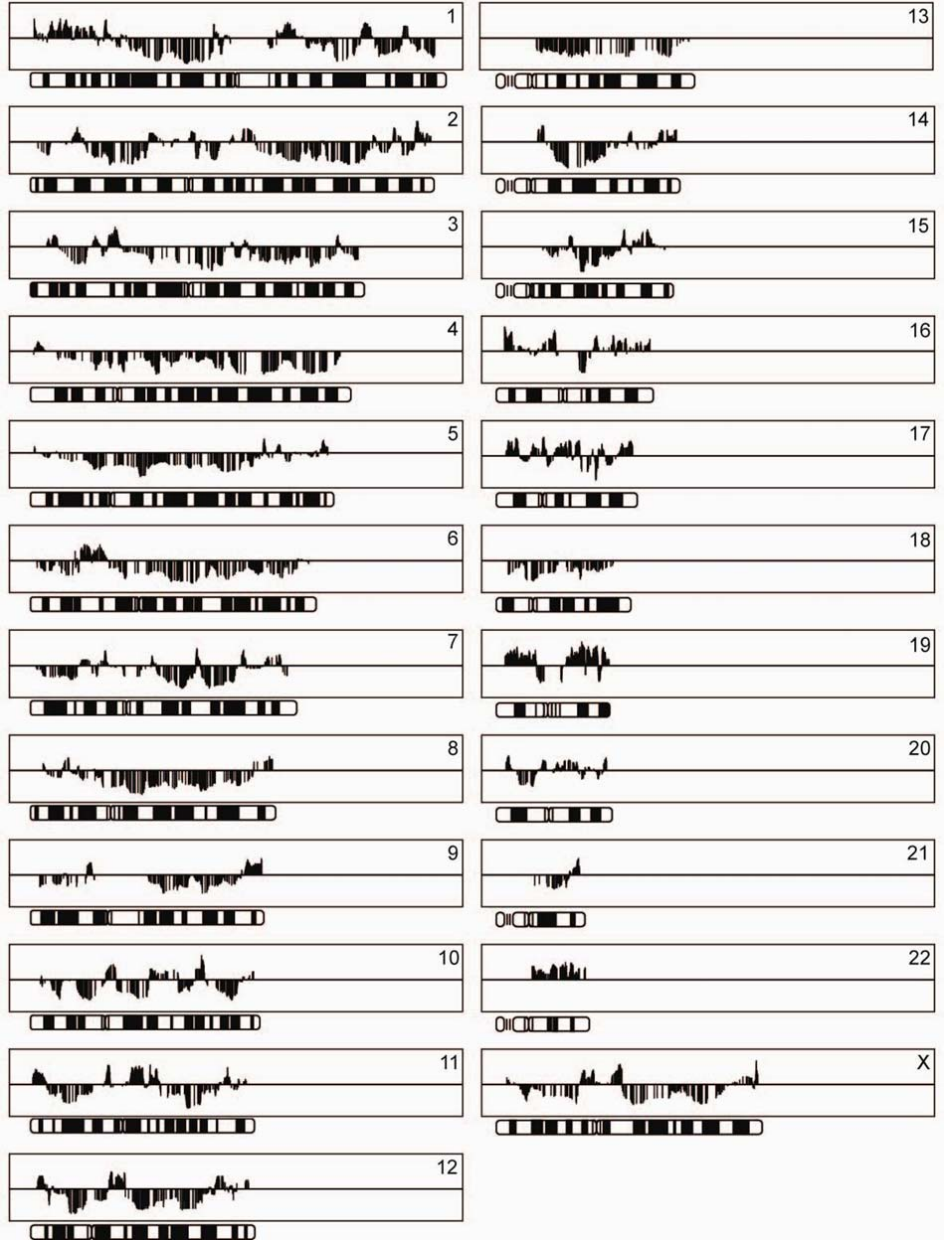
Regional factor loading profiles for 23 chromosomes. Factor loadings for TSR1 are shown on chromosomes as a moving median with a window size of 20 genes. Chromosome position is depicted on the *x*-axis and factor loadings for gene regions are given on the *y*-axis. Bars above or below the middle line represent inversely regulated chromosomal regions.

Next, in order to further characterize the transcriptional system regulation, we assessed whether genes similarly controlled by a specific TSR also cluster within chromosomal regions. Figure 5 shows regional factor loading profiles for TSR1. GSEA results in terms of chromosomal distribution of similarly controlled gene regions for other TSRs are available in the supplementary data. An application of a moving median with a window size of 20 genes clearly shows differences for the chromosomal regions. The Y-chromosome is not depicted as the number of genes in the dataset located on the Y-chromosome was less than 20, so a moving median could not be calculated. Several chromosomes (*e.g.* 1 and 11) have large regions of genes with predominantly positive factor loadings, interspersed with regions where genes have predominantly negative factor loadings. In contrast, chromosomes 4, 13 and 22 show hardly any regions of genes inversely controlled by TSR1, but, more remarkably, all these gene regions seem to be regulated in only one direction.

### Gene clustering based on TSR interaction

**Figure 6:**
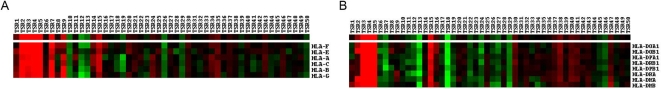
Heat map examples of gene clusters based on factor loadings with the first 50 transcriptional system regulators (TSRs). Each box in the heat map represents the factor loading of a gene with one of the TSRs (negative = red; positive = green). The first row shows the average factor loadings for the depicted cluster.

We clustered genes based on factor loadings with the first 50 TSRs in order to elucidate the dynamics of gene expression regulation, in which individual genes are classified into groups with similar regulation patterns. Gene clusters with distinct patterns of regulation were observed; clusters predominantly controlled by the first TSRs and clusters with a more diffuse pattern of TSR regulation (*e.g.* genes of which the products are involved in the biosynthesis of proteins from mRNA molecules). Furthermore, as functionally related genes often exhibit expression patterns that are correlated, we expected to observe clusters of functionally related genes based on TSR interaction. For example, Figure 6A and 6B show gene clusters with a strong biological relationship, *i.e.* the human leukocyte antigen system (HLA). All clustering results are available as supplementary data and can be depicted with Java TreeView, which can be downloaded from http://jtreeview.sourceforge.net/.

### Sample clustering based on TSR scores

**Figure 7:**
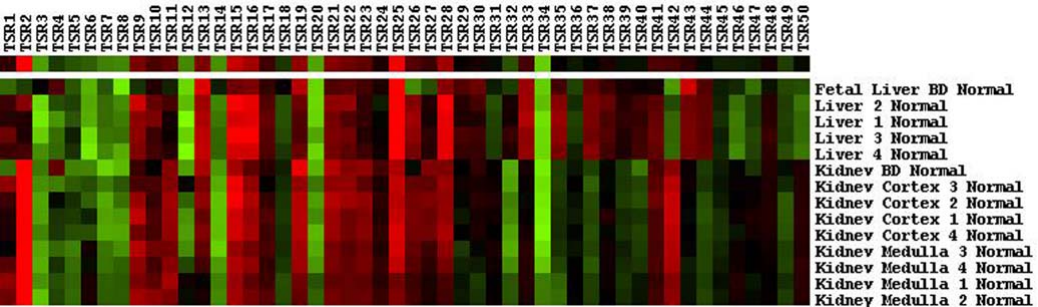
Heat map example of sample clusters based on transcriptional system regulator (TSR) scores. Each box in the heat map represents the score for a sample (negative = red; positive = green). The first row shows the average TSR scores for the depicted cluster.

Samples from the publicly available human body index were clustered to assess whether similar tissue samples have similar patterns in TSR scores. Clustering results for all samples are given in the supplementary data. Samples with identical tissue origins showed strong clustering, *e.g.* Figure 7 shows clustering of liver tissue samples as well as kidney samples.

## Discussion

Principal component analysis (PCA) on a large number of heterogeneous microarray experiments showed that a maximum of 50 statistically independent transcriptional system regulators (TSRs) can explain the vast majority of biological variance in gene expression in human as well as in mouse. Furthermore, we identified clusters of genes which expression is influenced in the same direction by specific TSRs and showed that gene expression can be conceptualized as a process influenced by a limited set of TSRs.

In microarray studies small sample sizes often present a major problem, such as overfitting, *i.e.* finding a discriminatory pattern by chance, which may occur when large numbers of genes are used to discriminate a small number of phenotypes.[7] Since a TSR is a weighted sum of genes, a TSR score can be calculated for each observed expression array. This data reduction allows us to characterize the expression profile of an individual microarray with 50 highly reliable TSR scores (TSR-profiling) instead of using thousands of individual genes (the software to compress expression array data in TSR scores is available at the supplementary website: http://129.125.155.240/tsr/). As an example we showed that similar tissues clustered together based on the first 50 TSR scores (Fig. 7). These results strongly suggest that the origin of tissues can be uncovered with the use of TSR scores. This could be applied for the identification of cancer metastases of unknown origin. The advantage is that a limited set of TSRs makes it is possible to analyze even small gene expression datasets with much less danger of false-positive results due to overfitting. Furthermore, TSR scores can be used to identify systematic changes of expression in gene clusters consisting of genes with small but systematic fold changes between phenotypes. This is important as we often do not know whether large fold changes in individual genes will have more biological relevance than smaller but coordinate fold changes in a set of genes.

Inherent to microarray experiments the measurement of gene expression is composed of biological signals and experimental noise.[8] We assumed that at least the first 50 TSRs, accounting for ∼70% of the information present in the entire dataset, capture the majority of relevant biological signals and that subsequent ones are likely to consist of noise and experimental artifacts, a principle which has also been described by Alter et al.[9] Normalizing gene expression data by filtering out these latter TSRs enables a meaningful comparison to be made of the expression of different genes across different arrays in different experiments (Fig. 6). Moreover, in addition to compare genes on the basis of their net expression, genes can now also be compared by their similarity in regulation of any chosen subset of TSRs. Therefore, the function of genes with a provisional status could be elucidated by looking at genes with known biological functions which are similarly influenced by our identified TSRs.

TSRs can also be linked to biological functions as we have shown a strong relation between TSR-related gene clusters and biological coherence in terms of functionality as well as cellular localization (Figures 2 and 3). Genes encoding for proteins located in the nucleus are influenced in opposite directions compared to genes encoding for proteins located in the plasma membrane. This might suggest that there is a regulatory process which could down regulate the expression of ‘nuclear’ genes when up-regulating ‘plasma membrane’ genes and vice versa. However, of note, a biological process represented by a GO ontology is not necessarily influenced by one specific TSR alone but can be influenced by more then one TSR; e.g. the contrast nucleus *vs.* membrane was seen in several TSRs. Although we do not know the nature of the transcriptional regulatory processes represented by our TSRs, this study provides insights into which specific biological processes are influenced in opposite directions. Further studies are needed to unravel the underlying nature and interplay of these TSRs.

Expression profiling of relevant disease tissues might help in candidate gene selection. However, such studies are often problematic, as relevant human tissue is hard to obtain. To overcome these sampling problems, the use of comparable mouse tissues seems a good option. Figure 4 shows a highly similar pattern of influence on gene expression between TSR1 in human and TSR2 in mice. Identical opposing GO ontologies are influenced in the same directions. Our results suggest a high homology between the transcriptional regulation of human and mouse. It is noteworthy that the first principal component from the PCA on the combined two-species dataset representing species differences revealed that GO ontologies such as primary metabolism and RNA processing were highly enriched. This is in line with expected metabolic differences between human and mouse. By filtering out the first principal component that represents expression differences between the species and/or platforms, will enable one with the remaining differences to translate mouse data to the human situation and *vice versa*, because species-specific variance in expression can be recognized and eliminated by subtraction.

So far several levels in transcriptional system regulation have been reported, *e.g.* classical DNA sequence regulators, epigenetic modifications, spatial and temporal organization of nuclear processes and chromosomes, organization of chromatin into higher-order domains, transcription factors and microRNAs.[10]–[15] These known levels in transcriptional system regulation may be represented by a combination of the TSRs identified in this study. In addition, some of the identified TSRs may represent other, as yet undiscovered levels of transcriptional system regulation. Interestingly, our work demonstrated that TSRs regulate genes from chromosomal regions predominantly in the same direction. This observation was most strongly pronounced for TSR1 (Fig. 5), suggesting it is strongly related to chromosomally related transcriptome regulation. A higher-order organization of transcriptome regulation in terms of chromosomal domains is also suggested by Caron et al.[16]

Our results give biological insights into regulation of the cellular transcriptome and provide a tool to characterize expression profiles with highly reliable TSRs instead of thousands of individual genes, leading to a >500-fold reduction of complexity with just 50 TSRs. This might open new avenues for those performing gene expression profiling studies.

## Materials and Methods

### Data acquisition

Publicly available microarray expression data of 17,550 human samples hybridized to HG-U133A or HG-U133 Plus 2.0 Genechips (Affymetrix, Santa Clara, California, USA) and 3,934 mouse samples hybridized to MG-U74A Genechip (Affymetrix, Santa Clara, California, USA) were obtained from the Gene Expression Omnibus.[17] These datasets contained a wide range of heterogeneous tissues (primary patient material, cell lines, normal tissues, etc.) and covered a multitude of different experimental conditions (transfected/transduced, stimulated or treated cells, etc.). For the human dataset, probes available on both platforms were selected for further analysis. Then the probes for the human as well as the mouse dataset were converted to official gene symbols, averaging log transformed expression values of multiple probes targeting the same gene. This resulted in 13,032 and 9,062 unique genes for the human and mouse datasets, respectively. Next, quantile normalization was applied separately to the log2 transformed expression values in each dataset.[18]

### Principal component analysis

Correlations between genes were calculated based on expression patterns across the diverse samples in both the human as well as the mouse datasets. Principal component analysis (PCA) was performed on the resulting correlation matrices, which is equivalent to factor analysis, leading to reduced dimensionality in gene space. We developed software to perform this task based on JAMA/C++, a translation of the Java Matrix Library, developed by the Mathworks and NIST, into C++ (http://math.nist.gov/tnt/). PCA is a method to condense a multi-dimensional dataset into a set of lower dimensions, in order to reveal the simplified linear structure of the data that often underlies it.[19] In this study PCA represents a transformation of a set of correlated genes into sets of uncorrelated linear additions of gene expression signals called principal components (PCs). PCs are able to summarize expression information and, for this application, can be interpreted as statistically uncorrelated transcriptional system regulators (TSRs).[9] In each TSR all genes are present but the weight of individual genes in the linear addition varies among TSRs. TSRs are constructed in such a way that the first TSR explains the largest amount of variance in expression and each subsequent TSR explains the largest amount of the remaining variance in expression while remaining uncorrelated with previously constructed TSRs. Since a TSR is a weighted sum of genes, TSR scores can be calculated for each observed expression array (TSR profiling). A TSR score can be seen as the degree of activity of the regulator in different cellular states or phenotypes. We provide a software tool capable of calculating individual TSR scores for observed expression arrays (see supplementary information online). Subsequently, the correlation between individual gene expression and TSR scores can be calculated (*i.e.* a factor loading). A factor loading can be seen as the amount of control a specific TSR has on the net expression of a particular gene. A high positive or negative factor loading with a TSR indicates that a gene's expression is strongly influenced by this specific TSR. Clusters of genes with contrasting factor loading signs (*i.e.* positive *vs.* negative) are inversely regulated by a specific TSR. For further reading on PCA we recommend a publicly available tutorial.[20]

### Reliability of transcriptional system regulators

To investigate whether our method gives results that depend on the presence of specific arrays, we randomly divided the human dataset into two equally sized sets and then generated new TSRs using PCA, each based on only half of the data. To assess whether these separately generated sets were comparable, we calculated Pearson correlations between the factor loadings with TSRs from the two separate sets.

Furthermore, to validate that the identified TSRs were not artifacts of gene selection, we applied the split-half method on each TSR.[21] As described above, a TSR is a weighted sum of genes and a so-called TSR score can be calculated for each sample. In the split-half method, the genes of a TSR score are split into two random partitions and the resulting TSR scores of both parts are correlated (Pearson). High correlation indicates that TSR scores can be reliably calculated and that information from different genes is indeed identical and indicative of the same underlying TSR score.

### Gene set enrichment analysis (GSEA)

To investigate whether our identified statistically uncorrelated TSRs are related to biologically related gene clusters represented by known GO ontologies we used GeneTrail, a software program recently developed by a German team (http://genetrail.bioinf.uni-sb.de).[22]. This web-based application scores a sorted list of genes with respect to their enrichment of functional categories.[23] For each TSR we ranked the genes according to ascending factor loadings, *i.e.* from most negative to most positive factor loading. A factor loading is the correlation between individual gene expression and a specific TSR score (degree of regulator activation). The ranked list of genes, of which some belong to a functional set *S*, is then processed from top to bottom. Genes at the top and bottom (*i.e.* genes with high negative and positive factorloadings respectively) are genes most strongly controlled by our defined TSR. Whenever a gene belonging to the functional set *S* is found, an enrichment statistic (ES) is increased by a certain amount, otherwise the ES is decreased. This ES is depicted in graphs showing whether the genes that comprise a functional set *S* are accumulated at the top (red graph) or bottom (green graph) of the ranked list (see Figs 2 and 4). The red *vs.* green graphs show the biological coherence of opposing regulated gene clusters controlled by TSRs, *i.e.* gene clusters with positive *vs.* negative factor loadings. The minimum and maximum of this ES are used to estimate the significance of the enrichment; the more significant a functional set *S* is, the more important a TSR is in regulating the expression of genes belonging to *S*. [22] To adjust for multiple testing problems, we tested for the false discovery rate (FDR) according to Benjamini and Hochberg's method.[24] A significance threshold of *P*<0.05 after FDR correction was maintained. Although GeneTrail reveals many biological categories, we focused our analysis on Gene Ontology (GO) and chromosomal location.[25] We analyzed 5,760 GO categories containing more than two genes and also assessed 24 chromosomes (including X and Y) for enrichment in co-regulated genes.

### Similarities in transcriptional system regulation between human and mouse

To assess the similarity of the structure in transcription regulation between human and mouse, we applied a PCA to a combined two-species dataset containing 3,934 mouse arrays and 3,934 randomly selected human arrays. Expression data of 6,610 orthologus genes between human and mouse was selected for this dataset. If the structure of transcriptional regulation is similar between human and mouse, we would expect a similar, limited number of TSRs to be needed to summarize the same amount of variance in total expression as seen in the PCA performed on human and mouse expression data separately.

### Mapping human and mouse TSRs

To map TSRs identified in 17,550 human and 3,934 mouse arrays separately, in order to assess similarities in regulation of biological processes between human and mouse, we selected the factor loadings of the first 50 TSRs for 6,610 identified homolog genes, based on similar gene symbol identifiers. Then we calculated Spearman rank correlations between factor loadings from human and mouse TSRs. High correlation between a human- and a mouse TSR indicates that these TSRs control identical gene clusters in human and mouse in the same way.

### Gene clustering based on gene-TSR correlation

In microarray experiments, gene expression is composed of biological signals and experimental noise.[8] In our model the first 50 TSRs capture most of the biologically relevant signals and subsequent TSRs capture noise and experimental artifacts. Clustering genes according to their factor loadings with the first 50 TSRs instead of net expression patterns, of which a part is experimental noise, might be a more robust approach and could give more insight into the dynamics of gene expression regulation, in which individual genes are classified into multiple groups of similar regulation. We used average linkage hierarchical clustering according to the Euclidean distance measure (square root of the sum of the squared differences in each dimension) by using the Cluster 3.0 software.[26]

### Sample clustering based on TSR scores

Since a TSR is a weighted sum of genes, a so-called TSR score can be calculated for each individual sample. To assess whether similar tissue samples have identical TSR scores, we applied average linkage hierarchical clustering according to the Euclidean distance measure on the first 50 TSR scores. We selected 621 samples, representing over 90 distinct tissue types from the publicly available human body index dataset from the Gene Expression Omnibus (Accession number: GSE7307).

## Supporting Information

Table S1 (17.09 MB XLS)

Table S2 (12.07 MB XLS)

Table S3 (0.02 MB XLS)

Figure S1 (1.14 MB tif)
