# Supplementary figures and images for: A New Perspective on Transcriptional System Regulation (TSR): Towards TSR Profiling
Source: PLoS One. 2008 Feb 20;3(2):e1656. doi: 10.1371/journal.pone.0001656 (PMC2250855; doi:10.1371/journal.pone.0001656)

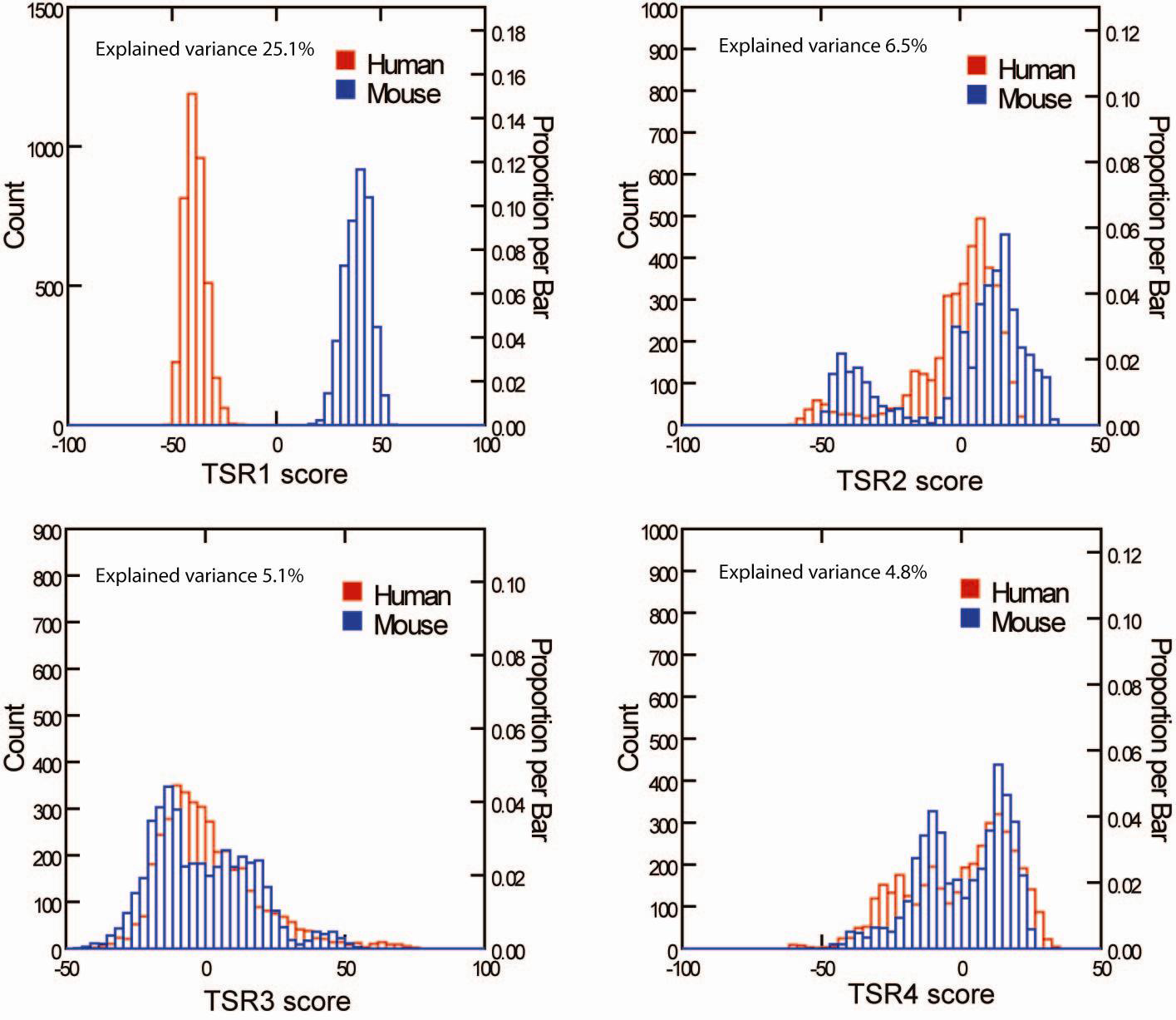

Supplement: Figure S1 — (1.14 MB tif) [file pone.0001656.s004.tif]
